# Emerging Role of Extracellular Vesicles in Embryo–Maternal Communication throughout Implantation Processes

**DOI:** 10.3390/ijms21155523

**Published:** 2020-08-01

**Authors:** Keigo Nakamura, Kazuya Kusama, Yoshihito Suda, Hiroshi Fujiwara, Masatoshi Hori, Kazuhiko Imakawa

**Affiliations:** 1 Laboratory of Veterinary Pharmacology, Graduate School of Agricultural and Life Sciences, The University of Tokyo, Tokyo 113-8657, Japan; knakam00@g.ecc.u-tokyo.ac.jp (K.N.); horimasa@g.ecc.u-tokyo.ac.jp (M.H.); 2 Department of Endocrine Pharmacology, Tokyo University of Pharmacy and Life Sciences, Tokyo 192-0392, Japan; 3 School of Food Industrial Sciences, Miyagi University, Miyagi 982-0215, Japan; suda@myu.ac.jp; 4 Department of Obstetrics and Gynecology, Graduate School of Medical Science, Kanazawa University, Kanazawa 920-8641, Japan; fuji@med.kanazawa-u.ac.jp; 5 Research Institute of Agriculture, Tokai University, Kumamoto 862-8652, Japan

**Keywords:** extracellular vesicles, conceptus, endometrium, implantation, progesterone, interferon tau, pregnancy

## Abstract

In ruminants, the establishment of proper conceptus–endometrial communication is essential for conceptus implantation and subsequent successful placentation. Accumulated evidence supports the idea that extracellular vesicles (EVs) present in uterine lumen are involved in conceptus–endometrial interactions during the preimplantation period. EVs make up a new field of intercellular communicators, which transport a variety of bioactive molecules, including soluble and membrane-bound proteins, lipids, DNA, and RNAs. EVs thus regulate gene expression and elicit biological effects including increased cell proliferation, migration, and adhesion in recipient cells. Uterine EVs are interactive and coordinate with ovarian progesterone (P4), trophectoderm-derived interferon tau (IFNT) and/or prostaglandins (PGs) in the physiological or pathological microenvironment. In this review, we will focus on intrauterine EVs in embryo–maternal interactions during the early stage of pregnancy, especially the implantation period in ruminant ungulates.

## 1. Introduction

In domestic ruminants, conceptus implantation to the uterine endometrium is a unique physiological process, consisting of blastocyst hatching, elongation, migration, apposition/attachment, and subsequent placentation [1]. The morula-stage embryo enters the uterus on days 4–6 postmating and then forms a blastocyst that contains an inner cell mass (ICM) and a blastocoel or central cavity surrounded by a monolayer of trophectoderm (TE). The blastocyst then hatches from the zona pellucida on day 8, and slowly grows into a tubular or ovoid form, which is termed a conceptus (embryo-fetus and associated extraembryonic membranes) [2,3]. The ovoid conceptus then begins to elongate into a filamentous form on days 12–13 in sheep or days 14–15 in cattle, respectively. During this period, elongating conceptus begins to produce a major cytokine, interferon tau (IFNT) [4,5,6]. IFNT prevents secretion of luteolytic pulses of prostaglandin F2-alpha (PGF2a) by uterine epithelium for the prolongation of the corpus luteum (CL) life span, which is a process described as the maternal recognition of pregnancy (MRP) [7,8,9,10]. After several days to a week of elongation, the ruminant conceptus occupies the entire length of the uterine horn ipsilateral to the CL, with extraembryonic membranes extending into the contralateral uterine horn, and begins its attachment to the uterine epithelium on day 16 in sheep and day 19 in cattle, followed by adhesion and placentation [11]. During this period, conceptus IFNT, together with maternal progesterone (P4) from functional CL, regulates endometrial gene expression, which sets up the uterine environment necessary for the establishment of conceptus migration, apposition, and initial attachment to the uterine epithelial cells in the ruminant species [12,13]. Thus, the establishment of proper conceptus–endometrial communication is required to allow conceptus implantation to the endometrium and subsequent maintenance of pregnancy.

The communication between different cell types is generally maintained through secretory soluble factors such as hormones and cytokines. In recent years, however, emerging evidence indicates that extracellular vesicles (EVs) produced by cells are also involved in cellular communication. In fact, EVs have been observed to transfer information to other cells, regulating cellular activities of the recipient cells [14]. Novel approaches and insights have made possible the extensive characterization of EVs, which contain surface receptors/ligands and cargo of proteins, lipids, metabolites, DNAs, and RNAs from the originating cells. Thus, EVs could be indicative of the cellular physiological state and function. In addition, the lipid bilayer of EVs made up of relatively high concentrations of cholesterol, sphingomyelin, ceramide, and detergent-resistant membrane domains, making these vesicles stable in extracellular spaces [15]. Following contact or uptake by recipient cells, EVs can regulate gene expression and elicit biological effects, including increased cell proliferation, migration, and adhesion. Evidence gathered indicates that EVs are produced by reproductive tissues/cells including follicular [16,17], oviductal [18], and endometrial cells [19], as well as in-vitro- and in-vivo-produced embryos [20,21,22]. Evidence is mounting that EVs of maternal or embryonic origin participate in the conceptus/fetal–endometrial interactions that are critical to pregnancy’s early stages, possibly continuing throughout the entire processes [23,24,25,26]. In this review, we will focus on the recent findings pertaining to EVs in embryo–maternal communication during the early stages of pregnancy, especially the implantation period.

## 2. General Concepts of EVs: Biogenesis, Secretion, and Cargo

In order to organize the data generated in different laboratories throughout the world, the International Society for Extracellular Vesicles (ISEV), which is the leading global professional society, constantly updates newly discovered EVs along with the available information. The documents entitled Minimal Information for Studies of Extracellular Vesicles (“MISEV”) Guidelines have been published and updated by the ISEV to provide information on better isolation and characterization of EV preparations, as well as suggestions to specific activities associated with EVs [27]. In this section, general information including subtypes and cargo of EVs based on the MISEV guidelines and the recent findings are provided to better understand the roles of EVs for conceptus–endometrial communication.

### 2.1. Subtypes of EVs

**Figure 1:**
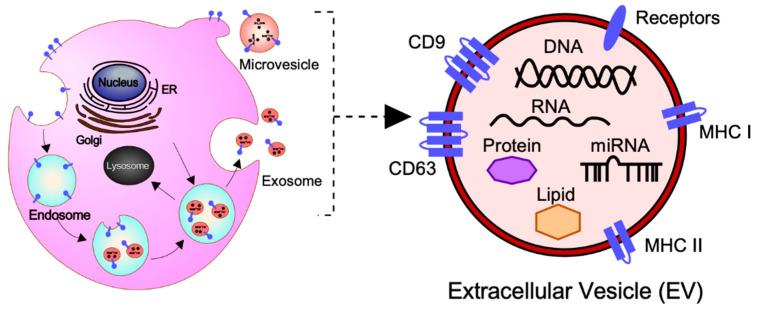
Biogenesis, secretion, and cargo of exosomes and microvesicles (MVs). Exosomes are small membrane-bound vesicles with a diameter of 50–150 nm, which are derived from endosomal multivesicular bodies. MVs are released directly from the plasma membrane with a diameter of 100–1000 nm into the extracellular space by budding and fission. Exosomes and MVs contain a variety of bioactive molecules including soluble and membrane-bound protein, lipids, metabolites, DNA, and RNA (mRNA, miRNAs, and other small regulatory RNAs), which regulate cellular activities of the recipient cells.

In general, EVs are defined as nano-sized, membrane-enclosed vesicles naturally released from the cells [27,28] and commonly classified as exosomes, microvesicles (MVs), and apoptotic bodies according to their sizes, biogenesis, and secretion (Figure 1) [29].

Exosomes are small membrane-bound vesicles with a diameter of 50–150 nm, which are derived from endosomal multivesicular bodies (MVBs). The formation is derived from the invagination of the plasma membrane (early endosome) and the subsequent fusion of endocytic vesicles mediated by the endosomal sorting complex responsible for transport (ESCRTs) and/or other components such as ceramides and tetraspanins [30,31]. Exosomes are the intraluminal vesicles secreted into the extracellular space by the fusion of MVBs with the plasma membrane.

MVs have a diameter of 100–1000 nm and are released directly from the plasma membrane into the extracellular space by budding and fission [30,31]. Their biogenesis mechanism is only a partially known; however, it has been found that MVs arise from the result of dynamic interplay between phospholipid redistribution and cytoskeletal protein contraction [32].

Apoptotic bodies are the largest vesicles with a diameter of 100–5000 nm, which originate from the fragmentation of the plasma membrane of cells undergoing the apoptotic processes [33]. Apoptotic bodies contain cell organelles, proteins, DNA fragments, and histones deriving directly from the intracellular environment. Apoptotic bodies are known to contribute to cellular waste management, unlike exosomes or MVs.

### 2.2. The Molecular Cargo of EVs

At present, the MISEV2018 guidelines suggest that three categories of protein markers should be demonstrated in all EV preparations for the presence of EVs (Categories 1 and 2) and their purity from common contaminants (Category 3) [27]. EVs transport a variety of bioactive molecules including soluble and membrane-bound protein, lipids, metabolites, DNA, and RNA (mRNA, miRNAs, and other small regulatory RNAs) [34]. Lipidomic analysis has shown that EVs, independent of their biogenesis, contain a multitude of lipids such cholesterol, sphingomyelin, ceramide, glycerophospholipids, phosphatidylcholine, and phosphatidylserine [27,35,36]. In addition, several proteomic analyses have shown that EVs contain different types of proteins, such as heat shock proteins (HSP70 and HSP90), major histocompatibility complex class I and II (MHC class I and II), tetraspanins (CD9, CD63 and others), endosomal sorting complex proteins required for transport (Alix and Tsg101) and chaperones, which are often used as protein markers [27,37,38]. Moreover, receptors including epidermal growth factor receptor (EGFR), membrane trafficking proteins (GTPases, Flotillin and Annexins), cytoskeletal proteins (tubulin and actin), and cytosolic proteins are also enriched in EVs. It should be noted that the cargo of EVs varies depending not only on their cellular origin, but also on their physiological and/or pathological condition.

## 3. Conceptus–Endometrial Communication Mediated by EVs during the Peri-Implantation Period

**Figure 2:**
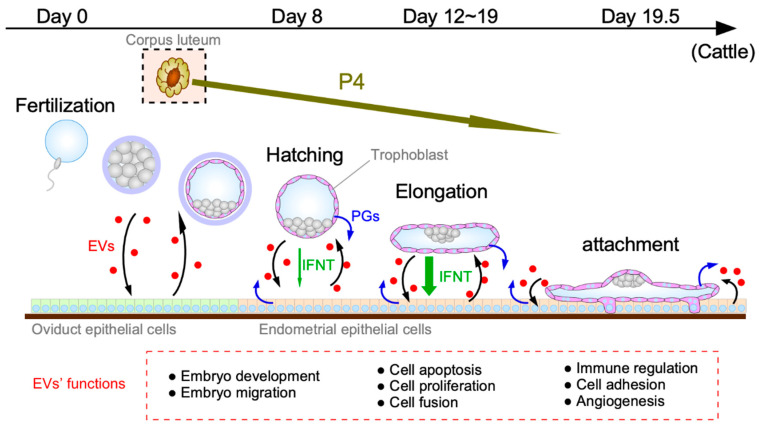
Roles of uterine extracellular vesicles (EVs) during the peri-implantation period. In domestic ruminants, the process of conceptus implantation to the maternal endometrium consists of blastocyst hatching, elongation, migration, apposition/attachment, and subsequent formation of the placenta. During these stages, EVs secreted by the conceptus and/or endometrium into the uterine microenvironment could have autocrine and/or paracrine biological effects on appropriate communication between the conceptus and the uterine endometrium. EVs are also interactive and coordinate with ovarian progesterone (P4), embryo-derived interferon tau (IFNT), and prostaglandins (PGs) for successful conceptus implantation and subsequent pregnancy establishment.

The establishment of mammalian pregnancy is dependent on successful implantation and placental formation, both of which require proper communication between conceptus and endometrium. It has previously been suggested that EVs are secreted from both the conceptus TE and maternal endometrium during the period of MRP in sheep [39]. Moreover, results from a human study suggested that exosomes could be released from the endometrial epithelium, thereby transferring molecular cargo to the blastocyst and/or the endometrium to promote implantation [40]. Uptake of embryo-derived EVs was observed in human primary endometrial epithelial and stromal cells [41]. These observations support the idea that conceptus–endometrial communications are mediated by EVs. In this section, we will introduce the role of EVs in autocrine and/or paracrine communications between blastocyst/conceptus and endometrium at different stages in the early pregnancy (Figure 2).

### 3.1. EV Functions during the Blastocyst Migration/Hatching Period

During the blastocyst migration/hatching period, secretions of the oviduct and uterus are involved in proliferation and development in preimplantation embryos. It is also noted that the early preimplantation mammalian embryo is relatively autonomous and can regulate its own development independently [42].

EVs exert biological effects on early embryonic development. One of the recent studies found that EVs were secreted from in-vitro-cultured bovine embryos of days 7–9, and the concentration of EVs was higher depending on the embryo’s competence [20]. Another bovine study has shown that the embryo-derived EVs improved the growth and viability of cloned bovine embryos and increased implantation rates as well as full-term calving rates [43]. In agreement with these observations, the porcine study demonstrated that parthenogenetic (PA) cloned embryos significantly improved blastocyst development rates of cocultured cloned embryos (nuclear transfer, NT) [44]. In this study, mRNAs for the pluripotency genes, *Oct4*, *Klf4*, and *Nanog*, were observed in PA embryo-derived EVs as well as cocultured cloned embryos, suggesting that the transfer of pluripotency genes via EVs could improve blastocyst development. Similar results were found in mice experiments, in which day 3–5 blastocysts microinjected with the Embryonic Stem (ES) cell-derived EVs before transfer into surrogate mothers significantly increased the likelihood of implantation [45]. This result indicates that ES cell-derived EVs improve the capability of TE cells within the blastocyst to migrate into the uterus and promote blastocyst implantation. Furthermore, another study found that miRNAs in EVs released during blastulation vary depending on the embryo’s quality, in which 12 miRNAs were upregulated and 15 miRNAs were downregulated in the good-quality blastocysts, respectively [46]. Thus, mammalian embryos secrete EVs into their surrounding environment, and these embryo-derived EVs, possibly together with endometrium-derived EVs, positively influence blastocyst formation, quality, and development in an autocrine and/or paracrine manner.

### 3.2. EV Functions Prior to Conceptus Attachment to the Uterine Epithelium

During this phase, the blastocyst/conceptus undergoes rapid morphological changes from spherical to tubular to filamentous forms and migrates freely throughout the entire lumen of the uterus. In domestic ruminants, this is the period when trophectoderms of the developing conceptus begin to secrete an antiluteolytic factor, IFNT, responsible for the maintenance of functional CL and form binucleate cells (BNCs). From the endometrial side, major morphological and functional changes through biological processes, including apoptosis and proliferation, are a prerequisite for uterine receptivity to conceptus implantation. The recent studies on uterine EVs find a variety of the cargo as well as the roles for biological processes during the period prior to conceptus attachment to the uterine epithelium.

Endogenous retroviruses (ERVs), integrated and abundant in the genomes of vertebrates, are involved in the formation of trophoblast BNCs. The sheep genome contains at least 27 copies of ERVs highly related to the exogenous and pathogenic Jaagsiekte sheep retrovirus (JSRV), which are termed endogenous JSRVs (enJSRVs) [47]. The enJSRV envelope genes, which are first detected on day 12, are abundant in the ovine reproductive tract, and integrated enJSRVs are packaged into endometrium-derived viral particles and transmitted to the TE and influence conceptus elongation and growth and development of the TE [48,49,50]. Recent studies on EVs provided evidence for packaging of enJSRVs within the EVs cargo between days 12 and 16 of gestation that could be delivered to heterologous cells in vitro [23,51]. These studies support the idea that enJSRVs, which regulate conceptus TE development including the differentiation of trophoblast BNCs from mononuclear TE cells beginning on day 14, could be delivered from the endometrium to conceptus TE via EVs.

Apoptosis is a typical form of programmed cell death by which tissues eliminate unnecessary cells. The occurrence of apoptosis has been described in many reproductive tissues including the uterine epithelium [52]. Available data from human pregnancy suggests that EVs induce apoptosis in activated immune cells [53]. Indeed, bcl-2-like protein 15 (BCL2L15), a regulator of apoptosis, was found in ovine uterine EVs during the periattachment period [22]. Furthermore, recent study in cows reported that EVs from day 17 of pregnancy increased expression of apoptosis-related genes, *BAX*, *CASP3*, *TNFA*, and *TP53* in primary endometrial epithelial cells (EECs) [54]. These results suggest that EVs induce the apoptosis of immune cells and EECs prerequired for conceptus implantation, during which a portion of the endometrial epithelium disappears.

Increased TE and endometrial cell proliferation is crucial for conceptus elongation and uterine receptivity. An in vitro study found that EVs derived from the ovine uterine lumen stimulated TE cell proliferation [23]. EVs purified from the porcine TE cells on day 12 pregnancy (the early implantation period) stimulated proliferation of maternal endothelial cells [24]. These studies indicate that EVs have biological effects that increase cellular proliferation for conceptus development and uterine morphological and functional changes.

Therefore, uterine EVs are involved in embryo–maternal communication through the modulation of biological processes including the differentiation of trophoblast BNCs, apoptosis, and cellular proliferation prerequisite for uterine receptivity to conceptus implantation.

### 3.3. EV Functions during the Conceptus Implantation Period

Noninvasive trophoblasts begin to attach to the uterine epithelium on day 16 in sheep and day 19 in cattle. This is the time when trinucleate and multinucleate cells, resulting from the fusion between trophoblast BNCs and uterine epithelial cells, begin to appear in the bovine and ovine uterine endometrium, respectively. Recent evidence on EVs supports the idea that the endometrial epithelium of the uterus as well as the TE of the elongating conceptus secrete EVs with biological effects, including immunomodulation and angiogenesis, required for successful conceptus attachment and adhesion to the uterine endometrium.

Vascular cell adhesion molecule (VCAM-1) is known as a cell adhesion mediator required for the establishment of the bovine conceptus adhesion to the uterine endometrium [55]. In investigating the effects of EVs and bovine uterine flushings (UFs) containing EVs on the adhesion molecule, UFs and intrauterine EVs obtained on days 20 and 22 postimplantation were observed to upregulate *VCAM1* transcripts in EECs [54,55]. In addition, proteomic analysis of purified human endometrial epithelial-derived exosomes treated with either estrogen or progesterone revealed that several members of the integrin family, which are essential for endometrium–embryo communication and implantation, were selectively packaged within endometrial exosomes [56]. These integrins within exosomes could be important for exosome docking to recipient cells and mediate trophoblast adhesion by interacting with ligands. Another investigation using mouse model revealed that exosome-associated miR-30d present in the endometrial fluid was transferred to murine embryos, resulting in overexpression of genes, *Itgb3*, *Itga7*, and *Cdh5*, which are involved in the embryonic adhesion to the maternal endometrium [57]. These results suggest that EVs play a major role in conceptus attachment and adhesion to the endometrium.

It should be noted that immunologic tolerance to the fetal allograft must be established to permit conceptus development and subsequent pregnancy maintenance. In the context of the immune and inflammatory responses during the conceptus attachment period, EVs appear to carry molecules potentially able to modulate the local endometrial immune system [58]. It is considered that their modulation occurs in order to stimulate or suppress the response depending on the receptors carried by the EV membrane and the chemical mediators in their cargos, such as proteins and miRNA. In addition, it was recently demonstrated that treatment of bovine EVs from day 20 of pregnancy, right after conceptus attachment is initiated, downregulated expression of immune-system-related genes in EECs [59]. Combinations of the miRNA profiles from bovine EVs and bioinformatics analysis predicted bta-miR-98 as a likely maternal immune system regulator. Additionally, placental exosome-derived bta-miR-499 was found to contribute to the regulation of local inflammation at the maternal–fetal interface by inhibiting NF-κB signaling, a key regulator of the inflammatory process. Therefore, inhibition of bta-miR-499 leads to inflammatory deregulation at the maternal–fetal interface and subsequent placental loss and fetal growth restriction [60]. These findings indicated that intrauterine EVs, especially miRNAs in EVs, contribute to regulating the maternal immune system for successful implantation.

Angiogenesis is an indispensable biological process of endometrial development, enabling firm attachment between the TE and the uterine luminal epithelium [61]. In pigs, placental formation is initiated during days 15–20 of pregnancy, which allows dramatic change in physiological processes, including angiogenesis, in the endometrial compartments [62]. A recent study in pigs found that endometrial cells and allantochorionic membrane cells from day 20 of pregnancy both released EVs [24]. These experimental data further revealed that porcine EVs isolated from both TE cells and maternal endothelial cells, indicative of the endometrial vasculature, contained abundant proteins and miRNAs, miR-126-5P, miR-296-5P, miR-16, and miR-17-5P, which may play a role in angiogenesis. Furthermore, another porcine study demonstrated that upregulated miR-150 in the umbilical cord blood derived exosomes enhanced the proliferation, migration, and tube formation of umbilical vein endothelial cells, whereas lower miR-150 levels in those exosomes exhibit intrauterine growth restriction [63]. These intensive studies have indicated that EVs are involved in the proliferation of the maternal endothelial cells and promote angiogenic processes, and suggest that EVs could become a possible novel approach for the treatment of disease related to aberrant angiogenesis during fetal development.

Consequently, these results support the notion that EVs mediate cell–cell communication through the biological processes including immunomodulation and angiogenesis required for successful attachment and adhesion of conceptuses to the maternal endometrium.

## 4. Roles of EV Interaction with Progesterone, IFNT, and Lipids, Including PGs

The establishment of pregnancy clearly requires complex physiological interactions between the conceptus and endometrium. Recently, accumulated evidence indicates that release of EVs into the uterine lumen by the elongating conceptus and the maternal endometrium is interactive and coordinated with ovarian P4, embryo-derived IFNT and embryo- and endometrium-derived prostaglandins (PGs), all of which are required for proper conceptus growth and implantation processes in the ruminant ungulates. In this section, we will focus on the newest findings related to the roles of EV interaction with P4, IFNT, and lipids, including PGs (Figure 2).

### 4.1. EV Interaction with Progesterone

The P4 secretion from functional CL is a prerequisite for the establishment and continuation of pregnancy in most mammals [64,65]. The actions of P4 on conceptus development are likely mediated by the endometrium, but little is known of how P4 contributes to conceptus survival and elongation. Recent findings in sheep provide novel insights into the biological effect of P4 on the production of EVs [66]. In this study, the presence of EVs were confirmed within the endometrial luminal and glandular epithelia from cyclic ewes, and the total number of EVs in the uterine lumen increased over two-fold with the P4 treatment in ovariectomized sheep. In addition, miRNA profiles from ovine endometrium and EVs from the uterine lumen followed by bioinformatics analysis revealed that P4 regulated seven miRNAs, of which four miRNAs were upregulated and three miRNAs were downregulated. Another study with human EECs found that P4 induce changes in the EVs production and protein cargo from EECs, and these EVs could increase the adhesive capacity of human blastocyst or mouse embryos [56]. Thus, these findings provide support for the idea that ovarian P4 directly and/or indirectly acts on endometrial epithelial production of EVs and their release into the uterine lumen. Further studies are needed to fully understand P4-regulated cargo in EVs and their function in pregnancy establishment.

### 4.2. EV Interaction with or without IFNT

Certainly, the trophectoderm of the elongating conceptus secretes IFNT, which itself regulates expression of elongation- and implantation-related genes in the endometrium and abrogates luteolytic mechanisms in ruminants [67]. Recently, several studies have indicated that IFNT interacts with uterine EVs over the course of the implantation processes. It was demonstrated that uterine EVs contained ovine or bovine IFNT [22,23,54] and increased the expression of several IFNT-stimulated genes *STAT1*, *STAT2*, *BST2*, *MX1*, *MX2*, and *ISG15* in bovine EECs during the periattachment period [22,54]. Furthermore, the treatment of IFNT stimulated the release of MX1 incorporated into exosome from ovine EECs [68]. In another bovine study, comprehensive miRNA analysis revealed that a total of 574 miRNAs, including 109 novel miRNAs, were detected in bovine EECs, from which 74 miRNAs were differentially expressed in those cells treated with IFNT [69]. Conversely, in vitro analyses in ewes indicated that intrauterine EVs also increased the production of IFNT protein by the conceptus TE cells in a dose-dependent manner [23,25]. Given that inert control liposomes did not increase IFNT in the culture media, these results indicate that the cargo of EVs contributes to IFNT production in TE cells.

It is generally accepted that conceptus IFNT greatly influences the endometrial transcriptome; however, the endometrium also responds to conceptuses in a manner independent of IFNT as well [70,71]. In fact, a recent study found that 82 transcripts in bovine EECs were uniquely induced by IFNT-independent intrauterine EVs, suggesting that uterine EVs also have biological effects on uterine receptivity in an IFNT-independent manner [26].

Thus, these findings support the notion that EVs present in the uterine lumen are involved in complex conceptus–endometrial interactions both independently and in conjunction with IFNT. Interestingly, it is likely that conceptus secretes not only IFNT itself, but also IFNT incorporated into EVs, both of which act on the uterine endometrium, respectively. It is well documented that blood contains EVs for organ-to-organ communications [72,73]. Recently, extrauterine or endocrine effects of IFNT have also been noted [74,75], suggesting that EVs including IFNT could directly or indirectly function on extrauterine tissues as well. However, it is clear that further in vivo evidence is needed to support the biological role of EVs and their potential interaction with IFNT required for pregnancy establishment.

### 4.3. EV Interaction with Lipids, Including PGs

EVs are also composed of lipids and may carry specific lipids, lipids metabolites, including PGs, and other enzymes for lipid metabolism that can modify the phenotype of recipient cells [76]. Most previous studies on EVs have mainly focused on roles for protein and RNA cargo of uterine EVs in embryo implantation. In a recent study, however, comprehensive lipid profiling of uterine EVs isolated from day 14 cyclic and pregnant sheep revealed that eight classes of lipids were included in cyclic and pregnant EVs, in which several lipid patterns differ significantly between EVs from cyclic and pregnant ewes, indicating that these populations of lipids are affected by pregnancy status [25]. In ruminants, a variety of PGs that are synthesized and secreted by both conceptus and endometrium have autocrine and paracrine effects on conceptus development, endometrial function, and endometrial responses to P4 and IFNT during early pregnancy [77,78,79,80]. It was recently found that prostaglandin synthase 2 (PTGS2), a rate-limiting enzyme in PG synthesis, was present in CD63- and HSP70-positive ovine EVs [51]. Furthermore, aldo-keto reductase family 1, member B1 protein (AKR1B1), a PG synthase, was found in uterine EVs obtained from days 15 and 17 pregnant sheep [22]. AKR1B1 has also been detected in pregnant bovine UFs containing EVs on day 16 [81]. Indeed, further experiments are needed to definitively define the biological role of lipids in uterine EVs, however, these results suggest that uterine EVs contain a diverse population of lipid cargo, including PGs, which may be responsible for conceptus elongation and implantation coordinated with actions of P4 and IFNT.

## 5. Potential Role of EVs for Clinical Application in Farm Animals

The embryo transfer (ET) industry has been growing rapidly. Indeed, ET data provided that a total of 1,129,041 bovine embryos and 17,868 ovine embryos were transferred commercially worldwide in 2018 [82]. It is generally accepted that the majority of bovine embryonic losses occur during the second and third weeks of pregnancy [83,84,85,86], suggesting that reducing embryonic losses at these stages result in higher productivity and economic efficiency in ruminants.

First, EVs may be of clinical significance for in-vitro-fertilized (IVF)-ET settings because selection of high-quality IVF blastocysts is required to increase successful IVF-ET rates. It was reported that EVs are secreted by in-vitro-cultured bovine embryos into culture media and their characteristics associated with embryo quality [87]. Human embryos secrete miRNAs, which could be packed in exosomes into culture media [88]. Notably, some of these miRNAs, including miRNA-191, were differentially expressed according to the fertilization method, chromosomal status, and pregnancy outcome. These findings suggest that cargo of EVs in the spent embryo medium could be biomarkers predictive of high-quality blastocysts, which would be a powerful noninvasive approach and improve IVF-ET successes.

Second, the application of EVs as biomarkers in early embryonic mortality or early pregnancy diagnosis could improve the rates of pregnancy successes as well. It is noteworthy that the cargo of EVs changes based on the extracellular environment. Comprehensive miRNA sequencing on serum EVs in day 17 pregnant and embryonic-mortality cattle identified that 27 miRNAs were significantly increased in day 17 embryonic mortality compared to those of the pregnant group [89]. Furthermore, miRNA profiles from EVs in the maternal blood of cattle on pregnant day 21 found that the somatic cell nuclear transfer-derived embryonic loss group exhibited lower abundance of 27 miRNAs than were found in successful pregnancy groups [90]. These observations indicate that pregnant and embryonic-mortality animals could be diagnosed through the detection of individual miRNA from the circulating EVs.

Given the observations that EVs secreted by the conceptus and/or endometrium are likely to promote conceptus implantation to the endometrium, third, potential clinical application would be to deliver specific cargo via EVs into the uterine cavity during the early pregnant stages. Recent studies have reported that the addition of bovine embryo- and uterus- derived exosomes increased the cleavage rate and blastocyst formation in cloned embryos [43,91], whereas the uterine exosomes derived from cows with endometritis significantly decreased the blastocyst formation rate of in-vitro-fertilized embryos compared to those derived from healthy cows [92]. These results provide support for the idea that the delivery of specific cargo via EVs into the uterine cavity conditions the uterine environment to result in better embryo development or endometrium receptivity for improved pregnancy success.

## 6. Conclusions

It is well documented that proper biochemical and cellular communication between the conceptus and the uterine endometrium are required for conceptus implantation and subsequent placentation. Recent progress suggests that uterine EVs may gain recognition as critical to conceptus–endometrial communication during the peri-implantation period in concert with the well-characterized molecules. It should be noted that uterine EVs could have autocrine and/or paracrine biological effects at different stages in the early pregnancy. As discussed earlier, uterine EVs are interactive and coordinated with ovarian P4, trophectoderm-derived IFNT, and/or lipids, including PGs, for conceptus elongation and conceptus implantation and subsequent placentation in the physiological or pathological microenvironment. Although significant details including the biogenesis, cargo, and definitive roles of uterine EVs have not yet been established, the rapid technological advances in the field of EV research will provide a strong impetus to clarify their effects on the peri-implantation processes.

In addition, EVs could become advanced tools for diagnosing and/or therapeutic agents useful to the reproductive field. For example, EVs in the spent embryo medium would be a predictive biomarker for selection of high-quality IVF blastocysts. In addition, the serum EVs following artificial insemination or embryo transfer would be a noninvasive biomarker for detecting pregnancy status. The last potential application would be to deliver specific cargo via EVs into the uterine cavity to improve embryo development or endometrium receptivity for more pregnancy success. Further research is required to characterize differences in the cargo of EVs between pregnant and nonpregnant or embryonic-mortality animals, which ultimately improves fertility rates in agriculturally important animals.

## Abbreviations

AKR1B1	aldo-keto reductase family 1, member B1
BCL2L15	bcl-2 like proteins 15
BNCs	binucleate cells
CL	corpus luteum
EECs	endometrial epithelial cells
EGFR	epidermal growth factor receptor
ERVs	endogenous retroviruses
ES	embryonic stem
ESCRTs	endosomal sorting complex responsible for transport
ET	embryo transfer
EVs	extracellular vesicles
ICM	inner cell mass
IFNT	interferon tau
ISEV	international society for extracellular vesicles
IVF	in vitro fertilized
JSRV	jaagsiekte sheep retrovirus
MHC	major histocompatibility complex
MISEV	minimal information for studies of extracellular vesicles
MRP	maternal recognition of pregnancy
MVs	microvesicles
MVBs	multi-vesicular bodies
NT	nuclear transfer
PA	parthenogenetic
PGF2a	prostaglandin F2-alpha
PGs	prostaglandins
PTGS2	prostaglandin synthase 2
P4	progesterone
TE	trophectoderm
UFs	uterine flushings
VCAM-1	vascular cell adhesion molecule
